# *Le devoir de mémoire*: Professor Didier Pittet’s reflections on an international career defying boundaries and expectations

**DOI:** 10.1017/ash.2023.508

**Published:** 2024-03-21

**Authors:** Didier Pittet

**Affiliations:** Geneva University Hospital, Geneva, Switzerland

Professor Didier Pittet is the former Hospital Epidemiologist and Director of the Infection Control Program & World Health Organization (WHO) Collaborating Centre on Patient Safety, University of Geneva Hospitals and Faculty of Medicine, Switzerland. He holds honorary professorships at Imperial College (London), Hong Kong Polytechnic University School of Health Sciences (Hong Kong) and the First Medical School of the Fu (Shanghai), and Doctorate Honoris Causa (UCL Louvain, 2021; University of Buenos Aires, 2023). Professor Pittet is also a fellow of the Royal Medical Association (Ireland) and Honorary Member of the All-Russian Scientific Societies of Epidemiologists, Microbiologists, and Parasitologists and the French National Academy of Medicine.

He has authored or co-authored more than 500 scientific publications and is editor of the textbook “*Hand Hygiene – A Handbook for Medical Professionals*” (Wiley-Blackwell, 2017). Professor Pittet is a member of the editorial boards of many prestigious scientific journals, including The Lancet Infectious Diseases, and is also an editorial consultant for The Lancet. The books “Clean Hands Save Lives” (Ed L'Âge d’Homme, 2014; new edition 2022, available in 19 languages) and “Adapt to Adopt” (Ed Nouvelle Imprimerie Laballery, 2021) written by French writer Thierry Crouzet, and the documentary film “Clean Hands” (Aftermedia 2016, 8 languages) describe Didier Pittet’s medical odyssey dedicated to the promotion of hand hygiene and patient safety worldwide.

Professor Pittet is the recipient of several national and international honors including a Commander of the British Empire awarded by Her Majesty Queen Elizabeth II for services to the prevention of healthcare-associated infection (HAI) in the UK (2007) and the Legion d’Honneur (France) awarded by President Emmanuel Macron (2022), as well as awards from the Society for Healthcare Epidemiology of America (2008), the European Society of Clinical Microbiology and Infectious Diseases’ Award for Excellence (2009), the American Society for Microbiology (2016), the Robert Koch Award (2017), the Pasteur Medal (2020), and the Ayliffe Award (2022).

Since 2005, Professor Pittet has been working closely with the WHO as the lead adviser of the First Global Patient Safety Challenge, whose Clean Care is Safer Care program is active in hospitals in 189 of the 195 UN member states. Professor Pittet conducted numerous research projects, regarding the epidemiology, prevention, and control of infections and the development of innovative strategies to improve hand hygiene compliance and the quality of patient care to promote their safety. He is also involved in numerous humanitarian projects. Professor Pittet is also credited with revolutionizing patient care processes in hospitals by replacing hand washing with soap and water with the systematic use of alcohol-based handrubs (ABHRs) and spreading this change in practice to healthcare centers around the world. His work has also involved the donation of a patent-free ABHR formulation to the WHO to facilitate its global distribution at lower cost, leading to its inclusion in the WHO list of Essential Medicines in 2012.

In June 2020, Professor Pittet was appointed by the President of the French Republic, Mr. Emmanuel Macron, as president of an independent mission to evaluate the management of the COVID-19 crisis and to better address the risks posed by the pandemic and deliver the report of this commission in May 2021.

The following is a transcript of a video interview with Dr. Pittet on October 26, 2023, edited for clarity.

## ASHE: Tell us about your unique training path that led you to infection prevention?

DP: I had an influential mentor who told me, “You need to do internal medicine, then, I want you to do infectious diseases,” but my plan was to do critical care, and he said, “No, no, no, you have to do infectious diseases.” This mentor, who was my “father in medicine,” is Professor Francis Waldvogel, who was the first physician to introduce the field of clinical infectious diseases to Switzerland. And so, after internal medicine and critical care, I completed training in infectious diseases and conducted basic science research on neutrophils at his recommendation.

At first, I said “no, no, no, I don’t want to do basic science research. I’m a clinician and I want to see patients,” but he insisted, and I ultimately did enjoy this experience. My mentor then said, “well, now we need to send you to a place for additional training so you can return to Geneva with something new for our institution.” I said “yes, why not?” To make a long story short, I visited Dick Wenzel in Charlottesville, which is a beautiful and charming place, but Dick said “don’t come now because I’m leaving for a sabbatical; I’m going to the UK and then I will leave Charlottesville for Iowa City, Iowa. So, you will have to meet me in Iowa.”

So, I moved to Iowa in 1989 and stayed until 1992. There, I had the privilege to work on his team of accomplished individuals like Loreen Herwaldt and Trish Perl. When I returned to Geneva, nobody was doing infection control. I oversaw the development of the infection control program, hiring a team of nurses to conduct surveillance and interventions. We had to understand what infections were and how many infections were occurring in our hospital. So, we set up a surveillance program and that is the story of the beginning of my career.

## ASHE: You’re best known for global promotion of hand hygiene. What events led to the use of ABHRs in patient care and what was the secret to the success of ABHRs?

DP: Well, it was in large part by chance. I came back from the US with a master’s degree underscoring the value of epidemiology applied to infection control. People in the hospital often ask, “20 francs,” or “$20 questions,” like “how frequently should we change the curtains?” “How frequently should we clean the floor?” But there are more important and basic questions, like “What are the rates of the major infections and solutions for these infections.” One of the first things I did in 1993 was set up a prevalence study of HAIs overseen by me and four infection control nurses. Our hospital was huge, with 2800 beds with a HAI prevalence rate of 18%, which was very high. So, I said, “okay, maybe we made a mistake… let’s do it again.” So, we repeated it several more times and found a prevalence rate of 16%. We realized that a major source of cross-transmission was likely the hands, so I said, “let’s look at hand hygiene,” which at the time involved washing with soap and water.

So, we developed a protocol emphasizing opportunities for hand hygiene, which ultimately became a compliance monitoring study. We realized that compliance with hand hygiene in the hospital was initially very low, around 40% for the hospital in general, about 3–4% in the emergency room, 20% in critical care, and so on. One very interesting observation was the strong relationship between the number of opportunities and compliance, meaning, the more opportunities, the lower the compliance. We published a series of articles in the Annals of Internal Medicine and others in 1999^1,2^.

Somehow, these findings were less surprising to our infection control nurses who understood that bedside nurses have no time for hand hygiene with soap and water, which took between 1-1.5 minutes. In the ICU on average, we observed 22 hand hygiene opportunities per hour, so, when you multiply 22 opportunities per hour by 1-1.5 minutes, you realize that you need at least 30 minutes for every hour of patient care just to wash your hands, which is totally impossible.

I recalled that ABHR existed and had used it in the microbiology lab for handling multidrug-resistant bacteria exposures. So, then I said, “why don’t we use this at the bedside?” However, at the time it only consisted of water and alcohol, which was very strong on your hands. So, I approached a friend in pharmacy who was a passionate about many things and said, “Let’s work on it, but let’s make sure it’s very well accepted.” Eventually, after trialing different solutions he prepared himself, we were ready to implement ABHR at the bedside as an easier and faster solution, which was as effective if not better than soap and water. However, this was a major cultural change. We needed to provide training and education and to monitor performance. So, I approached a university psychologist who was working on smoking cessation and convinced me that both involved behavior change and required a multimodal strategy. I worked with a cartoonist who would go to the wards, speak with healthcare workers, and draw things. We developed a promotional campaign team of the CEO, the medical director, the nursing director, and people from all hospital departments. Within three years we improved compliance from around 40% to almost 70%, and dropped HAI rates by 50%, including our methicillin-resistant *Staphylococcus aureus* rate by 80%. In academics they say “publish or perish” so we published the paper in the Lancet in 2000^4^, which even requested the posters we used on the wards. It was a lot of fun and led to several other publications^3–7^.

## ASHE: How did your close collaboration with the WHO develop?

DP: Following our ABHR experience, we received requests to visit our hospital from all over the world. Progressively people started implementing ABHR at their institutions, and slowly we were creating a world audience for patient safety. Sir Liam Donaldson, who was then Chief Medical Officer of England and introduced patient safety at the WHO, visited Geneva. That is how I came to help with the UK’s national campaign, as well as Belgium, Australia, and so on. Then Sir Liam approached me to lead the WHO’s first global Patient Safety Initiative for Hand Hygiene Promotion, which I accepted. We planned the program in 2004 and officially launched in October 2005.

## ASHE: Explain to us the genesis of the International Consortium for Prevention & Infection Control (ICPIC), now one of the leading infection prevention conferences worldwide? What were the major barriers and how did it become successful?

DP: Since my time with Dick Wenzel in Iowa City, I dreamt of creating a meeting of friends working collaboratively on the same topic, one that is science-based with the aim of sharing data before it’s published. When I came back to Geneva, I developed a global community of experts who loved to work on things like WHO guidelines. I wanted to grow this into a conference but had no money, so I approached the Chancellor of the University who helped me find a space. Then I called friends and sponsors to fund a small meeting every two years. We eventually moved the meeting to the United Nations International Conference Center for mission-based meetings. Having spent much time in resource-limited settings like Africa, I ensured that our meeting sponsors delegates from Africa to attend with all expenses paid. Part of the inaugural meeting was in French, and part was in English to accommodate everyone. Many health ministers from Africa attended the inaugural ICPIC, as well as the President of the Swiss Confederation. It gave a flavor to the meeting that was quite unique and legitimate. ICPIC is now held every two years, always in Geneva, Switzerland.

## ASHE: What is the future of infection prevention in the post COVID-19 pandemic era? What should we now focus on?

DP: Humanity has survived, but clearly, society and the economy were not adequately prepared. In infection control, we were not fully prepared and discovered things that we did not know about. You must constantly assess risk during pandemics, however, the prevailing critique during the 2009 Influenza H1N1 pandemic was that we “overreacted” leading some to say “during the next pandemic, we should not overreact.” When the COVID-19 pandemic started, the Chinese government was not open to showing us the real data, so we underestimated the situation.

Speaking of risk assessment and preparedness, can you imagine that more than 95% of masks were manufactured in China? Therefore, at first, we couldn’t get masks and medications during their lockdowns. The positive aspect of infection prevention is that CEOs who were not previously convinced about its importance now understand its role. COVID changed people’s impression of infection prevention, but I hope it will also change their views about preventive versus curative medicine. I’m only cautiously optimistic because many countries haven’t looked back on a very critical level and asked, “have we done well or not?”

To me, our global COVID-19 response was average due to a lack of preparation. I had the privilege to consult President Emmanuel Macron of France and to provide his government with a critique of their handling of the pandemic in comparison to other countries. I compared the pandemic management of most countries, and clearly no country did very well. Most countries did not even use their mandatory influenza preparedness plans because some argued that it was not the same virus. Fine, but at least 90% of it was in the plans, which nobody used.

The other important lessons are to leverage the pandemic for resources and encourage politicians to collaborate with the scientists and the scientists to collaborate with the politicians. The scientists should communicate the science, and politicians should use the science to make decisions. We need to approach these crises holistically and ask ourselves “what about the schools and the students? What about the economy and society?” Infection control must be included in these larger discussions.

## ASHE: What advice do you have for emerging epidemiologists and infectious diseases specialists?

DP: For me, it has been the best job you can imagine. In the beginning, it’s a job but then it becomes a mission and a passion. When you visit hospitals in Latin America, India, and Africa, and see that a simple strategy can be successfully implemented to reduce infections dramatically and save lives…there is no better feeling. We have estimated that our hand hygiene campaign is saving between 5M and 8M lives in the world per year. How can young doctors not be inspired to pursue infectious diseases and public health? To me, this is the most important selling point. It’s not a money-making job as we know, but in life, you should pursue your passions.

You should not only have fun but be effective and have a very holistic vision and be multidisciplinary. You must include EVERYONE, from the nurses to the people working in the cafeteria and cleaning the hospital. During COVID-19, I had to convince people in the hospital to get the vaccine. I did so by persuading them that they were part of a team that works for the benefit of the patient. If infection preventionists want to succeed, they need to understand this. You need to go to the bedside and connect with everyone to understand how best to implement protocols. Implementation science is key. In summary, my advice is to brace yourselves for a long and exciting journey!

## ASHE: Finally, what books are currently on your nightstand?

DP: I’m very interested in books that experts have written either during or after the pandemic to better understand why society failed in many ways, such as with the economy. For instance, what was the impact of religion or government action, and why was the uptake of the vaccine so different all over the world? What about the infodemic? How could we have done better? The world relied heavily on one region (Wuhan, China) as the major manufacturing hub of masks. As this region became the epicenter of the pandemic, access to critical equipment was crippled for the rest of the world. How could this vulnerability have been avoided?

Our duty now is to ensure we have adequately grown from the lessons of the pandemic using a global approach. In French, we call it *devoir de mémoire*, which means a duty to make memories. We need to understand why things like pandemics, political conflicts, and other pivotal moments in history have occurred and try our best to prevent them from happening again. Here is a list of books I’m currently reading in the spirit of *devoir de mémoire* to help make sense of all that transpired.

Jean-François Delfraissy, Claude Kirchner. Questions d'éthique au temps de la COVID-19. Avis du CCNE et du CNPEN, La Documentation Française, 2023

ISBN 978-2-11-157654-3

Emmanuel Hirsch. Une éthique pour temps de crise. Les Editions du Cerfs, 2022.

ISBN 978-2-204-15238-9

Emmanuel Hirsch. Pandémie 2020. Ethique, société, politique. Les Editions du Cerf, 2020.

ISBN 978-2-204-14190-

Tina Purnat, Tim Nguyen, Sylvie Briand (ed) Managing infodemics in the 21st Century- Addressing new public health challenges in the information ecosystem. Springer, 2023

ISBN 978-3-031-27791-7

Robert Sherertz, John Abramson. COVID Chaos. What Happened and why. World Scientific, 2023

ISBN 978-981-128-560-0

Craig Whitlock. The Afghanistan Papers. A secret history of the war. Simon & Schuster Paperbacks.

ISBN 978-1-9821-5900-9
